# Dengue Virus Nonstructural Protein 1 Induces Vascular Leakage through Macrophage Migration Inhibitory Factor and Autophagy

**DOI:** 10.1371/journal.pntd.0004828

**Published:** 2016-07-13

**Authors:** Hong-Ru Chen, Yung-Chun Chuang, Yee-Shin Lin, Hsiao-Sheng Liu, Ching-Chuan Liu, Guey-Chuen Perng, Trai-Ming Yeh

**Affiliations:** 1 The Institute of Basic Medical Sciences, College of Medicine, National Cheng Kung University, Tainan City, Taiwan; 2 Department of Medical Laboratory Science and Biotechnology, College of Medicine, National Cheng Kung University, Tainan City, Taiwan; 3 Department of Microbiology and Immunology, College of Medicine, National Cheng Kung University, Tainan City, Taiwan; 4 Department of Pediatrics, College of Medicine, National Cheng Kung University, Tainan City, Taiwan; University of California, Berkeley, UNITED STATES

## Abstract

Dengue virus (DENV) is the most common mosquito-borne flavivirus; it can either cause mild dengue fever or the more severe dengue hemorrhagic fever (DHF) and dengue shock syndrome (DSS). One of the characteristic features of DHF/DSS is vascular leakage; although DENV nonstructural protein 1 (NS1) has been proved to induce vascular leakage after binding to Toll-like receptor 4, the down-stream mechanism has not yet been fully understood. In the sera of DENV-infected patients, the concentrations of DENV NS1 and inflammatory cytokine macrophage migration inhibitory factor (MIF) are positively correlated with disease severity, but whether DENV NS1 induces vascular leakage through MIF secretion remains unknown. We demonstrated that recombinant NS1 induced vascular leakage and MIF secretion both in human endothelial cell line HMEC-1 and in mice. Furthermore, these phenomena were inhibited in the presence of anti-NS1 antibodies both *in vitro* and *in vivo*. DENV NS1 also induced LC3-I to LC3-II conversion and p62 degradation in endothelial cell line, which indicated the formation of autophagy. To clarify whether MIF or autophagy mediated DENV NS1-induced vascular leakage, various inhibitors were applied. The results showed that DENV NS1-induced vascular leakage and VE-cadherin disarray were blocked in the presence of MIF inhibitors, anti-MIF-antibodies or autophagy inhibitors. An Atg5 knockdown clone further confirmed that autophagy formation of endothelial cells was required in NS1-induced vascular leakage. Furthermore, DENV NS1-induced LC3 puncta were also decreased in the presence of MIF inhibitors, indicating that MIF mediated DENV NS1-induced autophagy. Taken together, the results suggest a potential mechanism of DENV-induced vascular leakage and provide possible therapeutic targets against DHF/DSS.

## Author Summary

Dengue is a viral disease transmitted by mosquitoes. The symptoms of dengue are often mild; however, severe dengue is one of the leading causes of hospitalization and death among children in Asian and Latin American countries. A symptom of severe dengue is vascular leakage, which can result in fluid accumulation, hypotension, circulatory collapse, and even death. For dengue and severe dengue, there is no specific treatment, and the only supportive treatment is to maintain a patient’s body fluids at normal levels. As a result, investigating the mechanism of how dengue virus (DENV) causes vascular leakage is an important and urgent issue. In this study, we demonstrated that DENV nonstructural protein 1 (NS1) induced vascular leakage through the secretion of macrophage migration inhibitory factor (MIF) and the formation of autophagy. Inhibition of MIF or autophagy formation effectively reversed NS1-induced vascular leakage both *in vitro* and in mice. These results provide possible therapeutic targets for treating vascular leakage in severe dengue.

## Introduction

Dengue virus (DENV) is the most common mosquito-borne flavivirus that spreads in tropical and sub-tropical areas. The World Health Organization estimates that more than 2.5 billion people, over 40% of the world’s population, are now at risk of dengue infection [1, 2]. DENV infection generally causes dengue fever (DF), which is often asymptomatic or results in a mild flu-like illness with intense joint pain and fever. However, a small proportion of cases develop into severe illness termed dengue hemorrhagic fever (DHF). DHF is characterized by vascular leakage, thrombocytopenia, and coagulopathy [3]. Among these characteristics, vascular (plasma) leakage results in hemoconcentration and serious effusions, which can lead to circulatory collapse and life-threatening dengue shock syndrome (DSS) [4, 5]. It has been estimated that there are 50–100 million infections and approximately 500,000 people with severe dengue requiring hospitalization each year globally. The mortality of DF is less than 1% with adequate treatment; however, severe disease carries a mortality rate of 26%. Despite the high mortality of DHF/DSS, there are still no effective drugs or vaccines available because of a limited understanding of the pathogenic mechanism [6].

DENV nonstructural protein 1 (NS1) is a 48 kDa glycoprotein that can be expressed on the cell surface as a dimer and secreted as a hexamer into the blood circulation of dengue patients. The NS1 hexamer is composed of three dimers, which forms a detergent-sensitive hydrophobic central cavity that carries a cargo of ~70 lipid molecules; the composition is similar to that of high-density lipoprotein [7–9]. The concentration of NS1 in the sera of DHF/DSS patients can reach 50 μg/ml, which is positively correlated with disease severity [10–12]. The secreted NS1 may bind to cell membranes via interactions with heparin sulfate and chondroitin sulfate [13]. NS1 can also interact with prothrombin to interrupt the coagulation cascade [14]. In addition, NS1 can activate complement to elicit complement-dependent cytotoxicity in endothelial cells or to escape from innate immunity attack [15–17]. Recently, NS1 has been shown to be able to induce vascular leakage via binding to Toll-like receptor 4 (TLR4) [18, 19]. Therefore, investigating the downstream effectors of NS1-induced vascular leakage may provide potential targets for treating DHF/DSS.

Vascular permeability is normally maintained by the well-regulated endothelial barrier structure, which plays a crucial role in the control of exchange of small solutes and macromolecules between the intravascular and interstitial space [20, 21]. The integrity of endothelial permeability is regulated by many factors. Under pathological conditions such as infection, vascular leakage may occur because of damage to endothelial cells or loss of endothelial barrier function [22]. The physical damage to endothelial cells can be a result of cell apoptosis, which will take time to repair. In contrast, dysfunction of the endothelial barrier is reversible and may occur because of exposure to various vasoactive mediators or cytokines leading to the disruption of cell-cell junctions [23]. Vascular leakage in DHF/DSS patients occurs on days 3–7 of the illness and will resolve within 1 to 2 days in patients who receive appropriate fluid resuscitation [24, 25]. Therefore, it is generally believed that a mechanism that induces vasoactive cytokines rather than structural destruction of endothelial cells may be the major factor responsible for vascular leakage in DHF/DSS [6, 26, 27].

In a previous study, we found that DENV infection can induce macrophage migration inhibitory factor (MIF) secretion, which can cause an increase in vascular permeability both *in vitro* and *in vivo* [28]. Using recombinant MIF, we further demonstrated that MIF induces endothelial hyperpermeability through autophagy and that this process is related to the degradation of junction proteins [29]. MIF is a 12.5 kDa protein that is widely expressed in different cells, including immune cells, platelets, hepatocytes, and endothelial cells. Under physiological conditions, MIF exists in cells as a trimer consisting of three identical subunits, resulting in a catalytic site located in the intermonomeric pocket. Under stress conditions, such as inflammation and hypoxia, MIF is secreted into the blood circulation to modulate both innate and adaptive immune responses [30]. Secreted MIF can bind to cell surface receptors such as CXCR2, CXCR4 and/or CD74 [31, 32], inducing downstream signals such as the phosphoinositide 3-kinase (PI3K)/Akt pathway or the mitogen-activated protein kinases (MAPK)/extracellular signal-regulated kinase (ERK) pathway [33, 34]. It is known that MIF secretion can also be induced upon TLR4 stimulation [35]. Therefore, it is possible that MIF-induced by DENV NS1 may play an important role in DENV-induced vascular leakage.

Autophagy is a degradation pathway that occurs when cells are under stress conditions such as starvation, hypoxia, and infection [36–38]. Autophagy begins with the sequestration of the area of the cytoplasm inside double-membrane vesicles called autophagosomes [39, 40], which subsequently fuse with lysosomes to form autolysosomes or late endosomes to produce amphisomes [41]. Two ubiquitin-like conjugation of autophagy proteins (Atg5 and Atg12) are essential for autophagosome formation. Atg5 and Atg12 promote lipidation of a cytosolic form of light chain 3 (LC3; LC3-I) to form the LC3-phosphatidylethanolamine conjugate (LC3-II). The lipidated LC3-II, which is tightly associated with the autophagosomal membranes, can be observed by immunofluorescence staining to monitor autophagy, in which LC3 puncta formation reflects the existence of autophagosomes. In addition, after fusion with lysosomes, adaptor protein p62 will be degraded in the autophagolysosomes. As a result, autophagy formation can be determined by the decrease of p62 or the increase of LC3-I to II conversion by immunoblotting analysis. It has been demonstrated that DENV infection promotes the formation of autophagy, which can enhance virus replication [42]. However, the role of autophagy in DENV-induced vascular leakage has not been studied. Therefore, we proposed and tested the hypothesis that dengue NS1 increases vascular permeability through MIF secretion and autophagy formation.

## Results

### DENV NS1 disrupted endothelial barrier function

**Fig 1:**
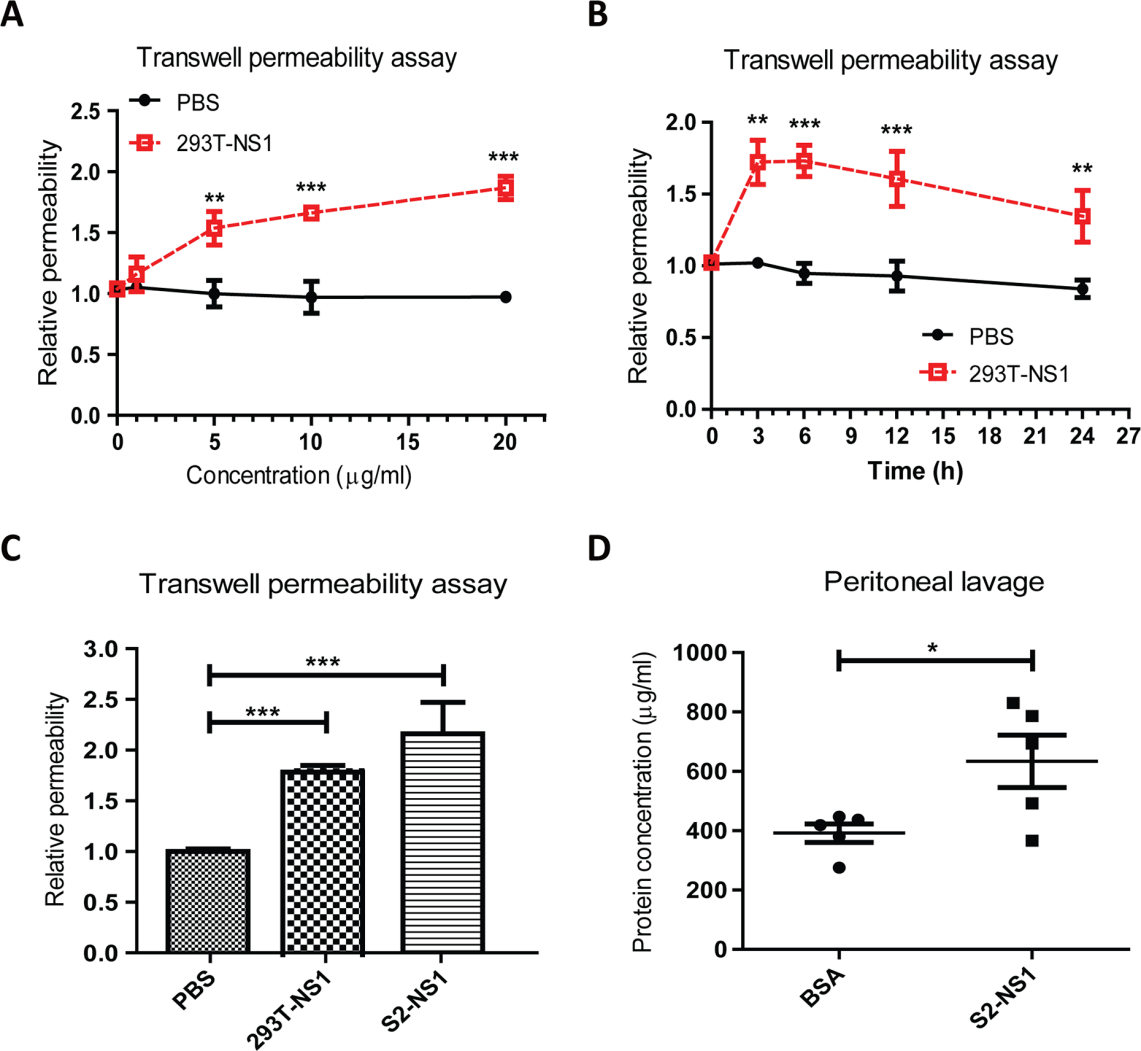
DENV NS1 increases the permeability of endothelial cells. (A) HMEC-1 cells were treated with different doses of 293T-NS1 for 6 h, and the endothelial permeability was determined by the transwell permeability assay. n = 4, triplicated. (B) HMEC-1 cells were treated with 20 μg/ml of 293T-NS1 or the same volume of PBS as the vehicle control for the indicated periods, and the endothelial permeability was determined by the transwell permeability assay. n = 3, triplicated. (C) HMEC-1 cells were treated with 20 μg/ml 293T-NS1 or S2-NS1 for 6 h, and the endothelial permeability was determined by the transwell permeability assay. The same volume of PBS was used as a control. n = 3, triplicated. (D) BALB/c mice were i.p. injected with 50 μg BSA or S2-NS1 for 6 h. After sacrifice, the abdominal cavity was washed with PBS, and the protein concentration in the peritoneal lavage was determined by the BCA method. n = 5, protein quantification was duplicated. *P<0.05, **P<0.01, ***P<0.001.

To assess the role of DENV NS1 protein in vascular permeability, recombinant serotype 2 DENV NS1 derived from human 293T cells (293T-NS1) and *Drosophila* S2 cells (S2-NS1) were used in this study. Different concentrations of 293T-NS1 were incubated with human endothelial cell line (HMEC-1) for 6 h and the endothelial permeability was determined by the transwell permeability assay. This result showed that 293T-NS1 increased endothelial permeability in a dose-dependent manner. At least 5 μg/ml of 293T-NS1 was required to increase the permeability (Fig 1A). The kinetic changes of 293T-NS1-induced endothelial hyperpermeability were also measured. Endothelial hyperpermeability was induced 3 h after incubating with 293T-NS1 (20 μg/ml), which persisted to 24 h (Fig 1B). Similar effects were found using S2-NS1 (Fig 1C). To determine whether NS1 caused vascular leakage *in vivo*, protein extravasation in the abdominal cavity of mice was measured 6 h after i.p. injection of bovine serum albumin (BSA) or S2-NS1 (Fig 1D). Protein concentrations in the abdominal lavages of S2-NS1-injected mice were significantly increased compared to those in BSA-injected mice, suggesting that S2-NS1 was able to induce vascular leakage in mice (Fig 1D).

### DENV NS1-induced vascular leakage was inhibited by anti-NS1 antibodies

**Fig 2:**
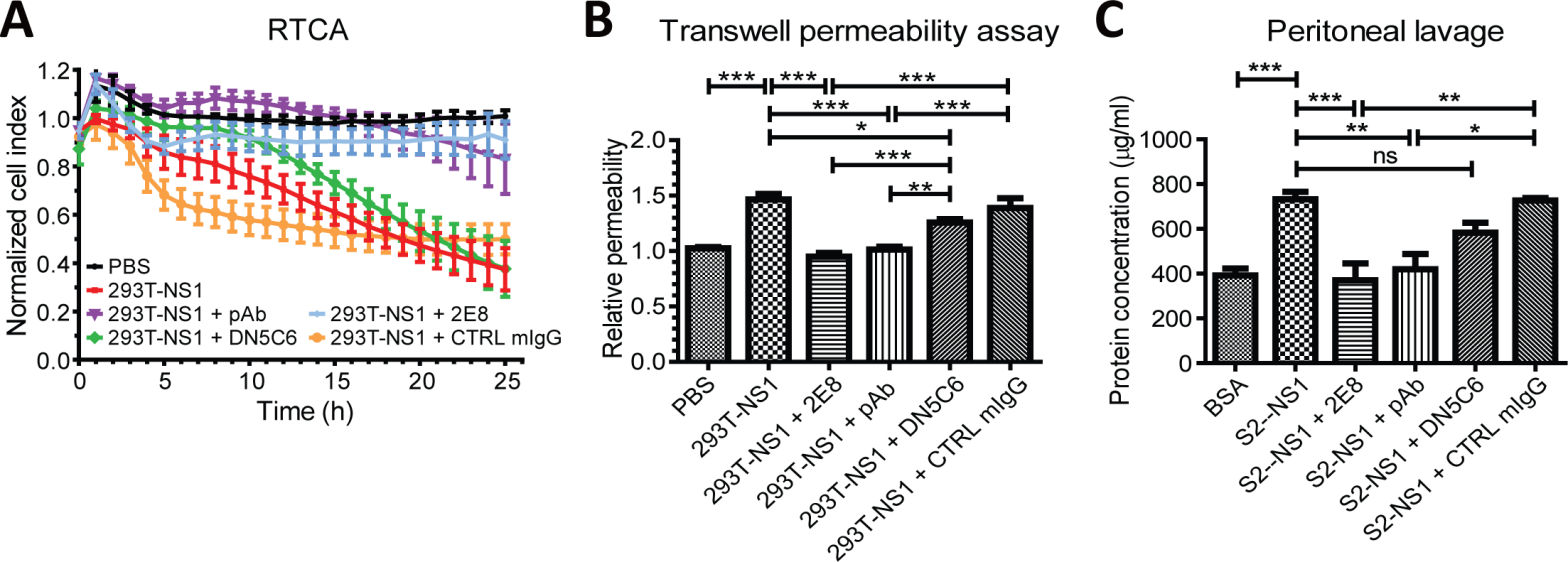
DENV NS1-induced vascular leakage is inhibited by anti-NS1 antibodies. (A) HMEC-1 cells were grown on a 96-well E-plate. When the cells grew to confluence, 293T-NS1 was added with or without anti-NS1 antibodies. PBS was added as a vehicle control, and CTRL mIgG was added as a negative control. The electrical resistance over a period of 24 h was measured and normalized as the cell index. n = 3, duplicated. (B) HMEC-1 cells were treated with PBS, 293T-NS1 with or without anti-NS1 antibodies. After 6 h, the endothelial permeability was determined by the transwell permeability assay. n = 4, triplicated. (C) BALB/c mice were i.p injected with PBS, S2-NS1 with or without anti-NS1 antibodies. After 6 h, the mice were sacrificed, and the abdominal cavities were washed with PBS. The abdominal lavage was collected, and the protein concentration was determined by the BCA method. n = 4, protein quantification was duplicated. *P<0.05, **P<0.01, ***P<0.001.

To confirm that the vascular leakage was specifically induced by NS1, we co-treated different anti-NS1 antibodies with 293T-NS1 and examined whether 293T-NS1-induced vascular leakage could be blocked. In addition, a real-time cell analysis (RTCA) system was used to monitor the kinetic change of endothelial permeability. These antibodies alone did not have any effect on the endothelial permeability of HMEC-1 cells either measured by RTCA or transwell assay (S1A and S1B Fig). However, 293T-NS1-increased endothelial permeability was inhibited in the presence of monoclonal antibodies (mAb) or polyclonal antibodies (pAb) against NS1 as measured by RTCA (Fig 2A). It was noted that different NS1 mAbs showed different blocking effect of which mAb 2E8 was better than mAb DN5C6 (Fig 2A). On the other hand, isotype control mouse IgG (CTRL mIgG) did not block 293T-NS1-increased permeability (Fig 2A). Similar results were also observed using the transwell permeability assay (Fig 2B). Likewise, *in vivo* experimentation also showed that anti-NS1 mAb 2E8 and pAb could block S2-NS1-induced protein extravasation in mice nearly to the basal value of the abdominal cavity, whereas CTRL mIgG could not (Fig 2C).

### DENV NS1 stimulated endothelial cells to secrete MIF

**Fig 3:**
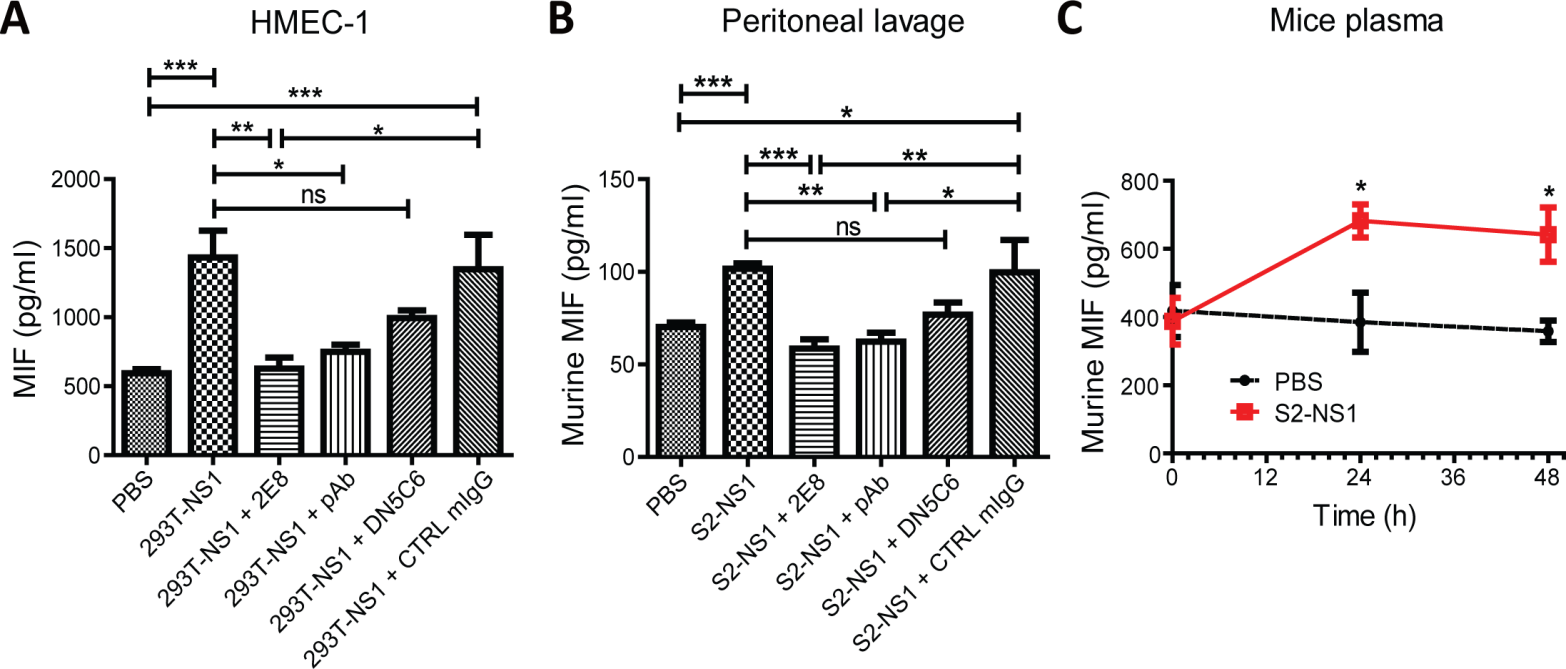
MIF secretion is induced by DENV NS1. (A) HMEC-1 cells were treated with PBS, 293T-NS1 with or without anti-NS1 antibodies for 6h. The supernatants of the cells was collected, and the MIF concentration in the supernatant was determined by ELISA. n = 3, MIF quantification was duplicated. (B) BALB/c mice were treated with S2-NS1 with or without anti-NS1 antibodies by i.p. injection. After 6 h, the peritoneal lavage was collected, and the murine MIF concentration in the peritoneal lavage was measured by ELISA. n = 5, protein quantification was duplicated. (C) BALB/c mice were i.v. injected with PBS or S2-NS1 for 48 h. Mice blood was collected by orbital sinus sampling at indicated time after i.v. injection. MIF concentration in the plasma was determined by ELISA. n = 3, duplicated. *P<0.05, **P<0.01, ***P<0.001.

To test whether MIF secretion was induced upon DENV NS1 stimulation of endothelial cells, the amount of MIF in the cell culture supernatant was determined by ELISA. As shown in Fig 3A, MIF secretion was induced by incubating 293T-NS1 with HMEC-1 cells. Anti-NS1 mAb 2E8 and pAb completely reversed 293T-NS1-induced MIF secretion, mAb DN5C6 showed partial inhibitory effect, while CTRL mIgG had no effect (Fig 3A). In addition, these antibodies alone did not alter the basal level of MIF secretion of HMEC-1 cells (S1C Fig). Similar to what we found in *in vitro* study, intraperitoneal or intravenous injection of S2-NS1 but not PBS into mice increased MIF concentrations in peritoneal lavage or plasma of mice, respectively (Fig 3B and 3C). Furthermore, anti-NS1 mAb 2E8 and pAb, but not mAb DN5C6 or CTRL mIgG, significantly inhibited S2-NS1-induced MIF secretion in mice (Fig 3B).

### Inhibition of MIF blocked DENV NS1-induced vascular hyperpermeability

**Fig 4:**
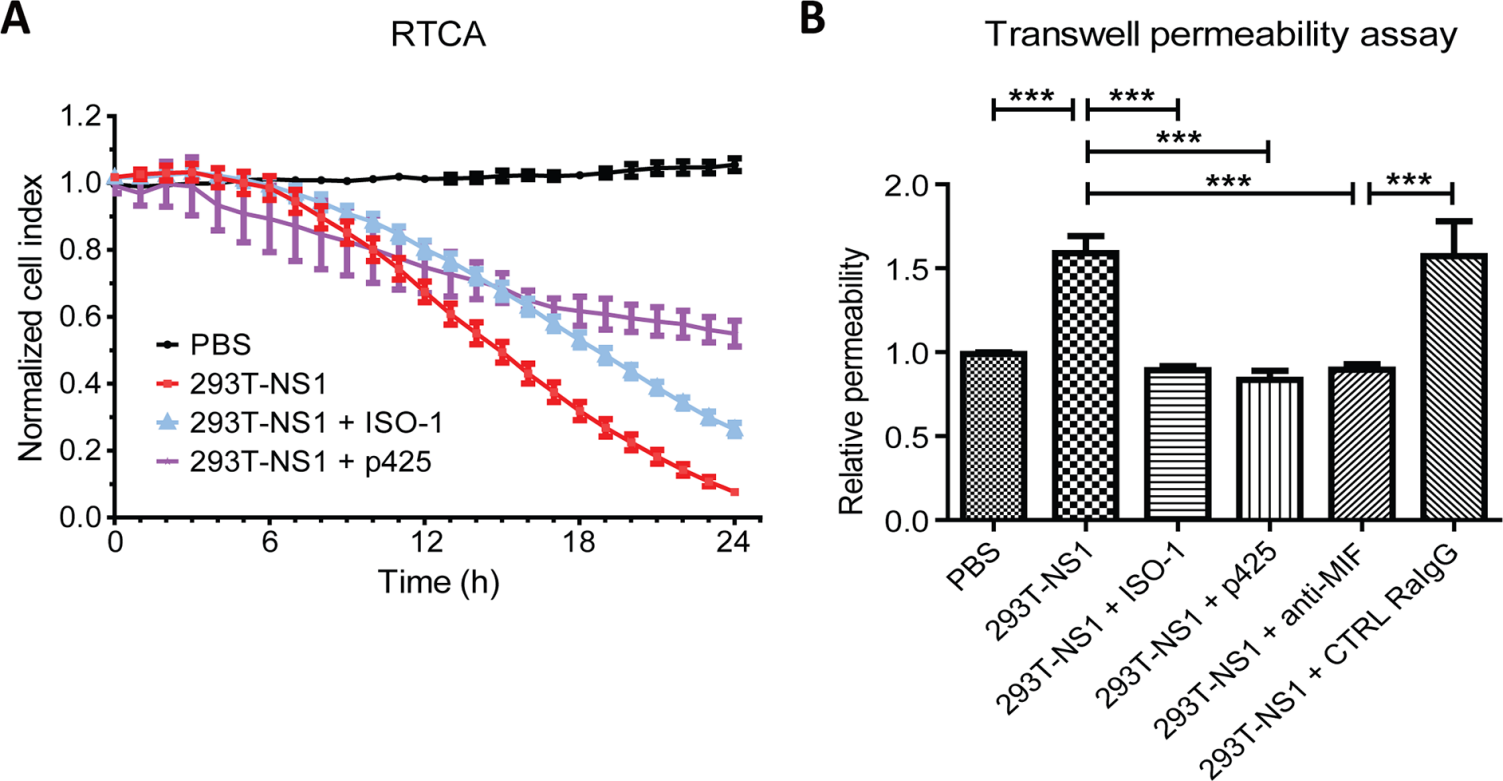
Inhibition of MIF prevents DENV-NS1-induced endothelial hyperpermeability. (A) HMEC-1 cells were grown on a 96-well E-plate. When the cells grew to confluence, 293T-NS1 was added with or without the MIF inhibitors. The electrical resistance over a period of 24 h was measured and normalized as the cell index. n = 3, duplicated. (B) HMEC-1 cells were treated with PBS, 293T-NS1, 293T-NS1 mixed with MIF inhibitors or 293T-NS1 mixed with anti-MIF pAb. CTRL RaIgG was also added as a negative control of anti-MIF pAb. After 6 h, the endothelial permeability was determined by the transwell permeability assay. n = 4, triplicated. ***P<0.001.

In our previous study, we found that MIF was involved in DENV-induced vascular leakage [28]; therefore, we tested whether inhibition of MIF could block DENV NS1-induced endothelial hyperpermeability. Inhibition of MIF by its inhibitors, ISO-1 or p425, decreased 293T-NS1-increased permeability as shown by RTCA (Fig 4A) and the transwell permeability assay (Fig 4B). In addition, anti-MIF pAb could also block 293T-NS1-increased endothelial permeability (Fig 4B). Rabbit IgG isotype control (CTRL RaIgG) was used as a negative control of anti-MIF pAb, which did not inhibit 293T-NS1-increased endothelial permeability. In addition, all these chemical inhibitors or antibodies alone did not have any effect on endothelial permeability, as shown in the supporting information (S1A and S1B Fig).

### DENV NS1-induced autophagy formation of HMEC-1 cells was inhibited by MIF inhibitor

**Fig 5:**
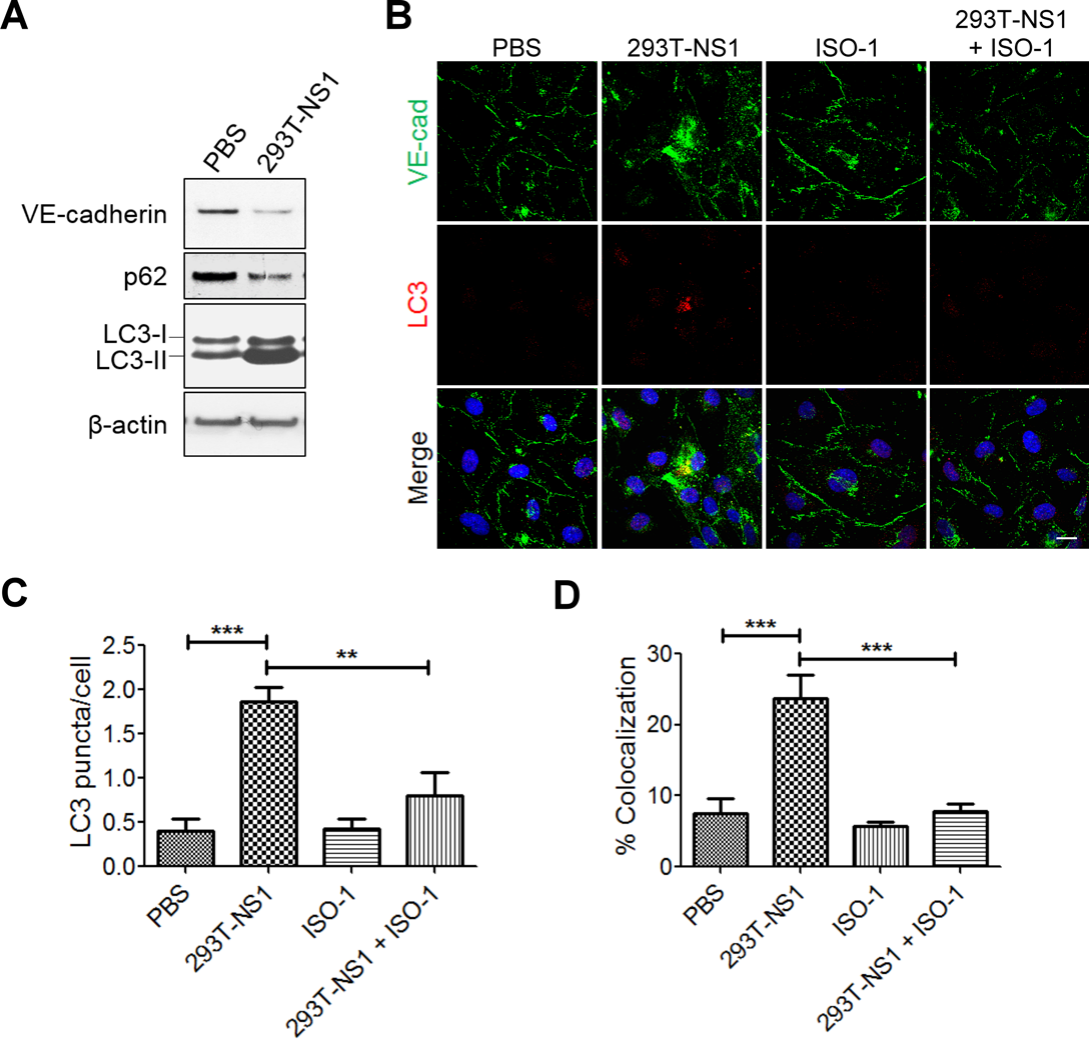
DENV NS1 induces autophagy formation of endothelial cells, which is subdued by MIF inhibitor. (A) HMEC-1 cells were treated with PBS or 20 μg/ml 293T-NS1 for 12 hours. The cell lysates were collected, and the relative protein levels of VE-cadherin, p62, and LC3 were determined by Western blot with specific antibodies. (B) HMEC-1 cells were treated with PBS, 293T-NS1, 293T-NS1 mixed with ISO-1, or ISO-1 only. After 6 h, the cells were fixed, and IFA was performed using specific antibodies. The number of LC3 puncta was quantified in (C), and the colocalization of LC3 and VE-cadherin was quantified in (D) by using FV1000 software. Bar: 10 μm. ***P<0.001.

MIF was reported to induce vascular leakage through autophagy formation [29], so we assessed whether DENV NS1 could induce autophagy of HMEC-1 cells. PBS- or 293T-NS1-treated HMEC-1 cell lysates were collected. Western blot analysis showed that 293T-NS1 induced p62 degradation and LC3-I-to-LC3-II conversion, which indicated autophagy formation in HMEC-1 cells (Fig 5A). Furthermore, 293T-NS1 also decreased the protein level of VE-cadherin, which might result in endothelial hyperpermeability (Fig 5A). Because the function of autophagy is to digest or degrade organelles or proteins, we wondered whether autophagy mediate DENV NS1-induced VE-cadherin degradation. Immunofluorescence staining was thus applied. Double staining of VE-cadherin and LC3 showed that the number of LC3 puncta was increased after 6 h of 293T-NS1 treatment (Fig 5B and 5C). In addition, cytosolic VE-cadherin colocalized with the LC3 puncta was found in 293T-NS1-stimulated HMEC-1 cells, indicating that some of the VE-cadherin proteins were embedded by autophagosomes (Fig 5B and 5D). Inhibiting MIF by its inhibitor ISO-1 decreased 293T-NS1-induced autophagy formation, LC3 puncta and the colocalization of LC3 puncta with VE-cadherin (Fig 5B–5D).

### DENV NS1-induced endothelial hyperpermeability was inhibited by autophagy inhibitors

**Fig 6:**
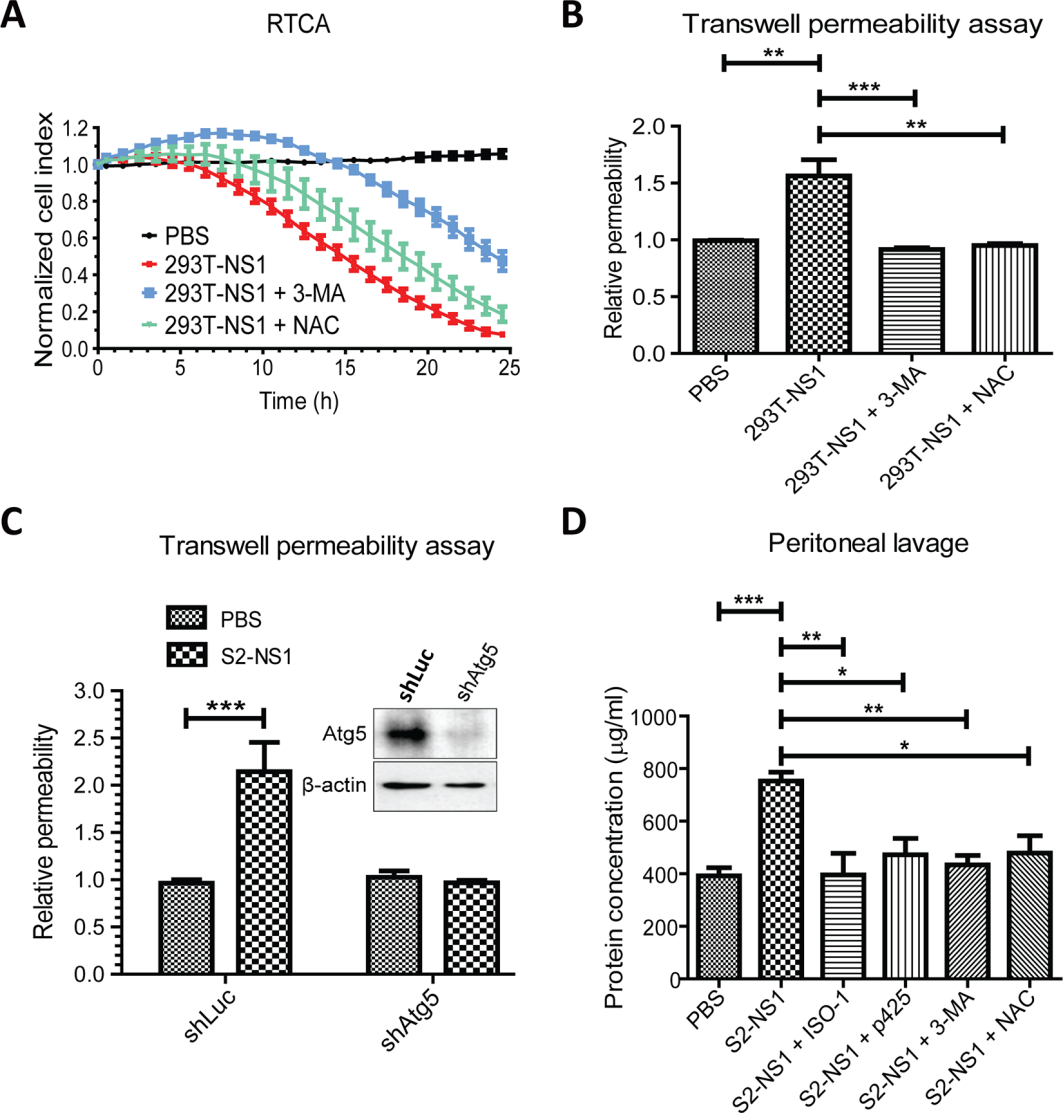
Inhibition of autophagy avoids DENV NS1-induced vascular leakage. (A) HMEC-1 cells were treated with PBS, 293T-NS1 or 293T-NS1 mixed with 3-MA or NAC for 24 h. The relative cell index was measured every hour with RTCA. n = 3, duplicated. (B) HMEC-1 cells were treated with PBS, 293T-NS1, or 293T-NS1 mixed with 3-MA or NAC for 6 h. The relative permeability was determined by the transwell permeability assay. n = 3, triplicated. (C) HMEC-1 cells were transfected with luciferase or Atg5 shRNA. After selection with puromycin, the resultant stable clones were treated with PBS or 20 μg/ml S2-NS1 for 6 h, and the endothelial permeability was determined by the transwell permeability assay with streptavidin-HRP and TMB. The knockdown efficiency is shown in the right panel with the results of Western blot analysis. n = 3, triplicated. (D) BALB/c mice were intraperitoneal injected with BSA, S2-NS1 with or without ISO-1, p425, 3-MA, or NAC. After 6 h, the mice were sacrificed, and the abdominal cavities were washed with 5 ml PBS. Protein concentration of the peritoneal lavage was quantified by BCA method. n = 3, duplicated. *P<0.05, **P<0.01, ***P<0.001.

To clarify whether autophagy mediated 293T-NS1-induced vascular leakage, autophagy inhibitors were used. RTCA results showed that 293T-NS1-induced endothelial hyperpermeability was inhibited by co-treatment with PI3K inhibitor 3-methyladenine (3-MA) or the reactive oxygen species (ROS) scavenger N-acetyl-L-cysteine (NAC) (Fig 6A). The results from transwell permeability assay also showed that both 3-MA and NAC inhibited 293T-NS1-increased endothelial permeability (Fig 6B), whereas neither 3-MA nor NAC alone had effect on endothelial permeability *in vitro* (S1A and S1B Fig). The importance of autophagy in NS1-induced endothelial hyperpermeability was further supported by the stable Atg5 knockdown HMEC-1 cells (shAtg5), which, unlike the control shLuc cells, were resistant to S2-NS1-induced endothelial hyperpermeability (Fig 6C). *In vivo* permeability assay was also applied to test whether inhibition of MIF or autophagy could rescue DENV NS1-induced vascular leakage in mice. The results showed that either inhibiting MIF or autophagy could rescue DENV NS1-induced vascular leakage in mice, indicating that both MIF and autophagy are involved in DENV NS1-induced vascular leakage (Fig 6D).

### NS1-induced VE-cadherin disarray of HMEC-1 cells was inhibited by MIF or autophagy inhibitors

**Fig 7:**
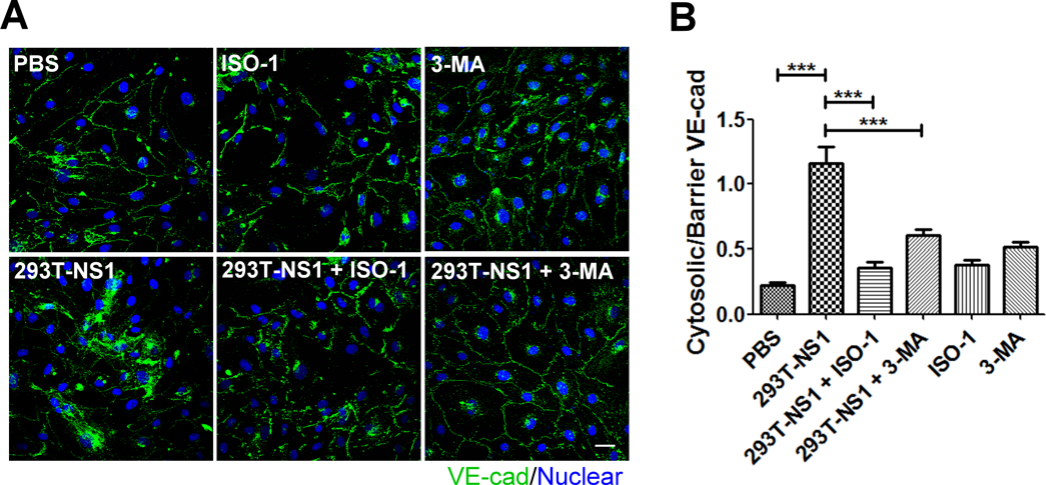
DENV NS1 induces VE-cadherin disarray, which is rescued by inhibiting MIF or autophagy. (A) HMEC-1 cells were treated with PBS, 293T-NS1, 293T-NS1 mixed with ISO-1, 293T-NS1 mixed with 3-MA, ISO-1 only or 3-MA only. After 6 h, the cells were fixed, and IFA was applied by anti-VE-cadherin antibodies. The fluorescence images were acquired by a FV1000 confocal microscopy. The fluorescent intensity was quantified by Image J software and cytosolic/perinuclear versus barrier/marginal VE-cadherin ratio is shown in (B) by measuring 50 cells for each condition. *P<0.05, **P<0.01, ***P<0.001.

Because MIF was previously shown to increase vascular permeability through the disarray of endothelial junction proteins ZO-1 and VE-cadherin, we sought to determine whether NS1 alters the alignment of endothelial junction proteins [29]. The immunofluorescence staining results showed that 293T-NS1 increased the ratio of cytosolic/barrier VE-cadherin of HMEC-1 cells (Fig 7A and 7B). To determine whether MIF and autophagy are involved in NS1-induced VE-cadherin disarray, we treated HMEC-1 cells with NS1 in the presence of MIF inhibitor ISO-1 or autophagy inhibitor 3-MA. The results showed that 293T-NS1-induced VE-cadherin translocation was inhibited in the presence of MIF or autophagy inhibitors and these inhibitors alone has no effects on VE-cadherin distribution (Fig 7A and 7B).

## Discussion

**Fig 8:**
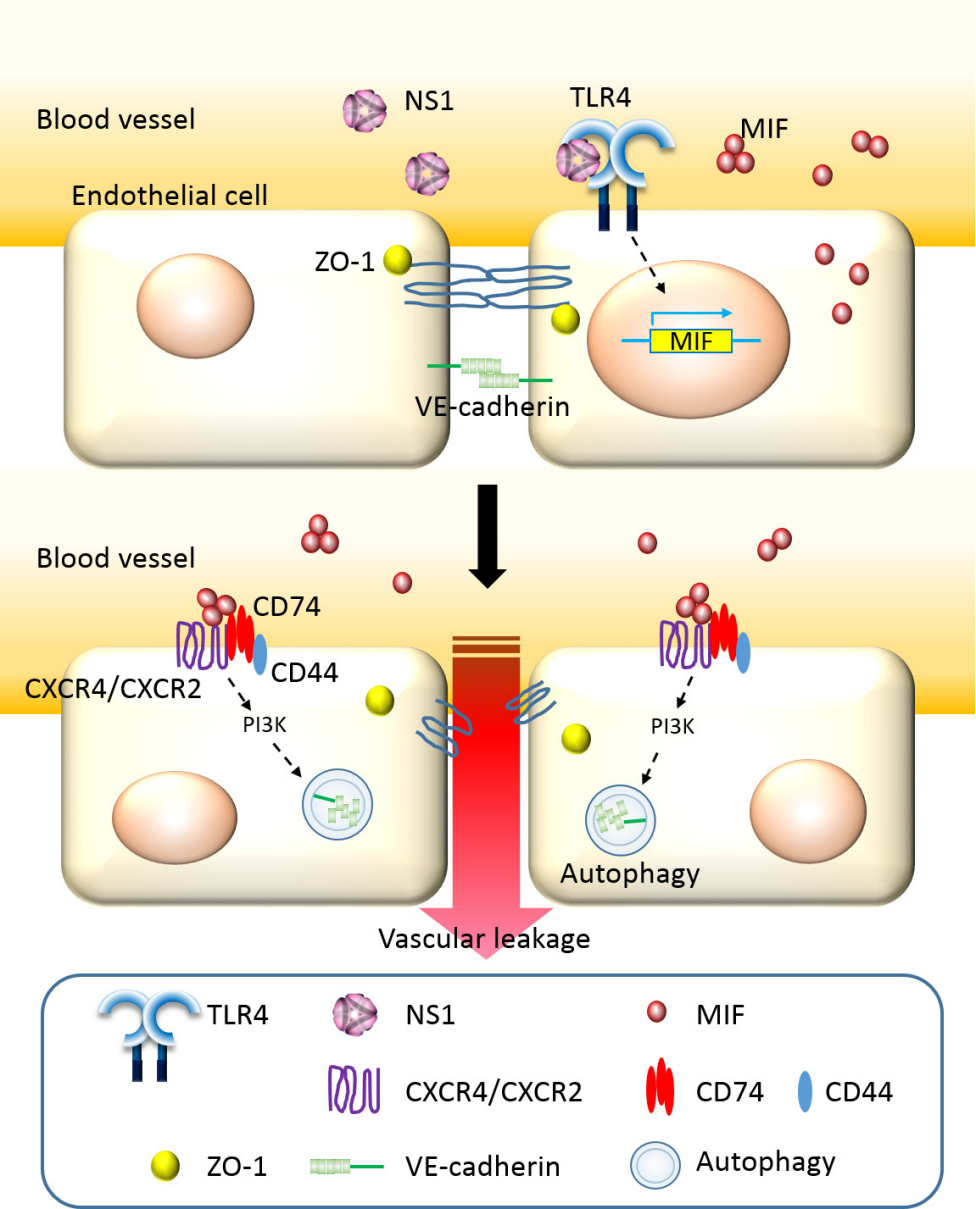
Hypothetical model of DENV NS1-induced vascular hyperpermeability. DENV infection increases the NS1 level in circulation. When NS1 binds to TLR4 on endothelial cells, the secretion and expression of MIF are induced. MIF then binds to receptor CXCR2/CXCR4 and CD74 on endothelial cells through paracrine or autocrine function. The receptor mediates the signal, which induces the formation of autophagy through PI3K. The autophagosomes mediate the degradation of junction proteins, which results in dysfunction of endothelial barrier and vascular leakage.

Little was known about the pathogenic roles of NS1 during DENV infection until recently. Two independent groups published papers which demonstrated that DENV NS1 can induce vascular leakage via TLR4 [18] and anti-NS1 antibodies or that NS1 vaccination can block this effect [19]. In this study, our results confirmed their findings and further suggests that MIF-induced autophagy of endothelial cells may mediate NS1-induced vascular leakage. The hypothetical model of the pathway by which DENV NS1 increases vascular permeability is shown in Fig 8.

In this study, we found that 5 μg/ml 293T-NS1 was sufficient to induce endothelial hyperpermeability at 6 h (Fig 1A). In *in vivo* experiments, we injected 50 μg S2-NS1 into mice. Because the total blood volume of a mouse is approximately 2–3 ml, the sera concentration of NS1 in mice is approximately 20 to 25 μg/ml. Furthermore, because the serum concentration of NS1 in dengue patients is estimated to range from 0.01 to 50 μg/ml [10], our experiments mimic the pathological condition in dengue patients. Even though further study is required to understand the contribution of NS1 in vascular leakage of dengue patients, these results suggest that NS1 can directly bind to endothelial cells to cause vascular leakage in dengue patients.

To further understand the interaction between NS1 and endothelial cells, we used different NS1 antibodies to block its effect. It is known that NS1 can also induce pathogenic antibodies that can cross-react with endothelial cells and induce endothelial cell apoptosis through molecular mimicry [43, 44]. Some of these anti-NS1 antibodies can also recognize platelets, resulting in thrombocytopenia [45]. Other anti-NS1 antibodies can cross-react with thrombin and plasminogen, resulting in inhibition of thrombosis and enhanced fibrinolysis [46]. Therefore, we used two different anti-NS1 mAbs 2E8 and DN5C6. Both of which did not bind to endothelial cells. We found that anti-NS1 mAb 2E8 showed better effect than mAb DN5C6 to block the activities of 293T-NS1 and S2-NS1 to stimulate endothelial cells. Similar results were also found by Beatty et al. which demonstrated that not all anti-NS1 antibodies can inhibit NS1-induecd vascular leakage [19]. Therefore, certain regions of DENV NS1 are more important for NS1 to interact with endothelial cells to induce vascular leakage. Identification of these regions may shed light to generate antibodies or vaccines to block NS1-induced vascular leakage.

It is known that MIF knockout mice show lower hemoconcentration and lethality compared with normal mice during DENV infection [47]. In sepsis, knockout or inhibition of MIF also increased survival rate of mice [48–50]. Previously, we demonstrated that MIF could mediate DENV-induced junction disarray and increase permeability in endothelial cells [28]. In this study, we further demonstrated that MIF is involved in DENV NS1-induced vascular leakage. Inhibition of MIF by its inhibitors can prevent DENV NS1-induced vascular leakage both *in vitro* and in mice. It is known that in addition to endothelial cells, other cells such as peripheral blood mononuclear cells (PBMC) can secrete MIF during DENV infection. Therefore, MIF secretion can be induced by either DENV infection or NS1 stimulation of different cells in dengue patients. However, in addition to MIF, other cytokines may also contribute to vascular leakage during DENV infection. Modhiran *et al*. showed that the expression of several cytokines including IL-6, TNF-α, IL-8, and MCP-1 were up-regulated in PBMC after DENV NS1 stimulation [18]. Many of these cytokines can also increase endothelial permeability [51–54]. Furthermore, culture supernatants from DENV-infected macrophage can induce endothelial cell apoptosis which is blocked by anti-TNF-α antibodies [55]. Even though it is known that MIF can augment the secretion of TNFα and counteracts the anti-inflammatory action of glucocorticoids [56, 57], DENV-induced vascular leakage may involve different mechanisms and the importance of MIF as therapeutic target against DENV-induced vascular leakage should be further studied.

It is known that autophagy is induced by DENV to prevent cell death and enhance viral replication during infection in human hepatoma cell lines [42, 58, 59]. Autophagy not only provides isolated environment but also provides energy and materials required for DENV replication by regulating lipid metabolism [60]. In addition, recent study showed that autophagy plays an important role in the antibody-dependent enhancement response in Fc receptor-bearing cells [61]. However, the role of DENV-induced autophagy in endothelial cells has not yet been discussed extensively. It has been reported that DENV NS4A is able to induce autophagy [62], but whether NS1 can also induce autophagy has not yet been reported. In this study, we showed that DENV NS1 induced autophagy, which mediated NS1-induced vascular leakage. As autophagy is required during DENV infection, inhibition of autophagy may prevent vascular leakage as well as suppress DENV replication. However, further studies are required to validate the therapeutic effects of autophagy inhibitors as anti-DENV drugs.

Taken together, our results suggest NS1-induced MIF secretion and autophagy may represent potential therapeutic targets for preventing vascular leakage in DHF/DSS. Our study highlights DENV NS1 as an important pathogenic factor in DHF/DSS. NS1-induced MIF secretion and autophagy may contribute to vascular leakage in DHF/DSS. Even though NS1 purified from DENV-infected cells or patients should be used to further confirm the pathogenic effects of NS1 on endothelial cells in the future, NS1-induced vascular leakage may represent a disease model in mice to develop potential therapeutic drugs and vaccines against dengue [63–66].

## Materials and Methods

### Ethics statement

All experiments were performed in conformity with the Guide for the Care and Use of Laboratory Animals (The Chinese-Taipei Society of Laboratory Animal Sciences, 2010) and were approved by the Institutional Animal Care and Use Committee (IACUC) of National Cheng Kung University (NCKU) under the number IACUC 99057.

### Cells

HMEC-1 cells were cultured in Medium 200 (Thermo Fisher Scientific, Waltham, MA) supplemented with 10% fetal bovine serum (FBS; HyClone Laboratory, Logan, UT) at 37°C in a 5% CO_2_ atmosphere. Stable clones of luciferase (Luc)-knockdown HMEC-1 cells were generated by a lentivirus-based short hairpin RNA (shRNA) system (National RNAi Core Facility, Academia Sinica, Taipei, Taiwan) targeting sequence 5’-GCCACAACATCGAGGACGGCA-3’. A stable clone of Atg5-silenced HMEC-1 cells was a kind gift from Dr. Chiou-Feng Lin. Both the shLuc and shAtg5 HMEC-1 cells were selected with 2 μg/ml of puromycin (MDBio, Inc., Taiwan).

### Recombinant proteins

In this study, we used two different commercialized recombinant NS1 proteins which were expressed in non-bacterial systems. Mammalian recombinant DENV serotype 2 NS1 protein, 293T-NS1 (The Native Antigen Company, UK), was engineered and expressed in the human 293T cell line. Another recombinant DENV serotype 2 NS1 protein, S2-NS1 (CTK biotech, San Diego, CA), was expressed in *Drosophila* S2 cells. Recombinant NS1 proteins were tested for endotoxin contamination by the Limulus amebocyte lysate (LAL) assay using the LAL Chromogenic Endotoxin Kit (Thermo Fisher Scientific, Waltham, MA) and shown to be endotoxin-free. Background endotoxin concentration of 0.036 EU/ml was found in 20 μg/ml 293T-NS1 and 0.018 EU/ml in 20 μg/ml S2-NS1, respectively.

### Preparation and purification of anti-NS1 antibodies

In this study, BALB/c mice were purchased from and maintained at the Laboratory Animal Center of NCKU. Purified recombinant DENV2 NS1 was used to immunize 6- to 8-week-old female BALB/c mice at a dose of 50 μg as previously described [67]. The first dose was administered in complete Freund's adjuvant (CFA), and the following three doses were administered in PBS. After sacrifice, mice splenocytes were fused with FO cells (Taiwan Medical Cell and Microbial Resources). The resultant hybrid cells were selected in hypoxanthine-aminopterin-thymidine medium. An ELISA was performed to screen for the specific antibodies against NS1. After the hybridoma clones 2E8 and DN5C6 were established, the hybridoma cells were i.p. injected into pristine-primed BALB/c mice to produce monoclonal antibodies in ascites. The antibodies were then purified using a Protein G column (GE Healthcare). Rabbit polyclonal anti-NS1 antibodies were purified from purchased recombinant DENV2 NS1-immunized antiserum (GeneTex, Inc., Irvine, CA). Endotoxin concentrations in these antibodies were also measured by LAL assay. Endotoxin concentrations in 30 μg/ml mAb 2E8, mAb DN5C6 and CTRL mIgG (Leadgene Biomedical, Taiwan) were 0.082, 0.092 and 0.028 EU/ml, respectively. Nevertheless, none of these antibodies alone could alter endothelial permeability nor induce MIF secretion as shown in the supporting information (S1 Fig).

### Inhibitors and treatment

In the *in vitro* experiments, 20 μg/ml (~ 400 nM) 293T-NS1 or S2-NS1 was applied. In the *in vivo* experiments, 50 μg S2-NS1 was applied by i.p or i.v. injection. Different anti-NS1 antibodies (mAb 2E8, DN5C6 and pAb) were used to block recombinant NS1-induced effects. The concentration of antibodies utilized in the studies was at 30 μg/ml (~ 200 nM) for *in vitro* experiments (Fig 2A, 2B, 3A and S1 Fig), and at 40 μg per mouse for *in vivo* setting (Figs 2C and 3B). Since IgG has two antigen binding sites, it can bind to more than one antigen by binding identical epitope carried on the surfaces of these antigens. Therefore, the amount of IgG antibodies used in current studies should be able to bind to most of the NS1 (~ 400 nM) we added for *in vitro* experiments. To inhibit MIF activity, the MIF tautomerase inhibitor (S,R)-3-(4-hydroxyphenyl)-4,5-dihydro-5-isoxazole acetic acid methyl ester (ISO-1) (50 μM; Calbiochem, La Jolla, CA) and 6,6'-[(3,3-Dimethoxy[1,1'-biphenyl]-4,4'-diyl)bis(azo)]bis[4-amino-5-hydroxy-1,3-napthalenedisulphonic acid] (p425)(100 μM; Calbiochem) were mixed with 293T-NS1 or S2-NS1 before treatment. Polyclonal anti-MIF antibody was purified using a protein G column (GE Healthcare), and 30 μg/ml was used to block MIF in 293T-NS1-treated cells. The endotoxin concentration in 30 μg/ml anti-MIF antibody and CTRL RaIgG (GeneTex) were 0.065 EU/ml and 0.02 EU/ml as determined by LAL assay, respectively. To inhibit autophagy, 5 mM of 3-MA (Sigma-Aldrich, St. Louis, MO) or 5 mM of NAC (Sigma-Aldrich) was used.

### *In vitro* permeability assay

To measure the permeability of endothelial cells *in vitro*, we used two different methods in this study: the transwell permeability assay and real-time cell analysis (RTCA) [68]. For the transwell permeability assay, cells (2 x 10^5^) were grown on a Transwell insert (0.4 μm; Corning B.V. Life Sciences, The Netherlands) until a monolayer was formed. The upper chambers were reconstituted with 10% FBS-containing medium with 293T-NS1, S2-NS1 and the inhibitors. At the indicated time points, the media in the upper chambers were changed to 300 μl of serum-free media containing 4.5 μl streptavidin-horseradish peroxidase (HRP) (R&D Systems, Minneapolis, MN). The medium (20 μl) in the lower chamber was collected 5 min after adding streptavidin-HRP and was assayed for HRP activity by adding 100 μl 3,3',5,5'-tetramethylbenzidine (TMB) substrate (R&D Systems). Color development was detected by a VersaMax microplate reader (Molecular Devices, Sunnyvale, CA) at 450 nm.

RTCA was used to test cell-cell or cell-matrix adhesion by detecting the electric resistance of the monolayered endothelial cells. High resistance indicates strong endothelial barrier function. Using this device allowed us to detect the kinetic changes in endothelial permeability. For RTCA, 1 x 10^4^ HMEC-1 cells were grown on a 96-well E-plate to form a confluent monolayer. After 293T-NS1 and the inhibitors were added, the resistance of the monolayer was recorded by an xCELLigence Real-Time Cell Analysis System (Cambridge Bioscience, UK) for 24 h.

### Vascular leakage in the peritoneal cavity

The method for testing vascular leakage in the peritoneal cavity was described previously [28]. Briefly, 8- to 12-week-old BALB/c mice were injected intraperitoneally with 50 μg of S2-NS1, which was solubilized in 500 μl of PBS with or without the inhibitors. The mice were sacrificed 6 h after the treatments and the abdominal cavity was washed with 5 ml PBS after sacrificing the mice. The concentration of protein in the abdominal lavage was determined using the BCA method (Pierce Biotechnology, Rockford, IL). Mean concentration was calculated with 3 to 5 mice in each condition.

### Enzyme-linked immunosorbent assay (ELISA)

The MIF concentration in the cell culture medium was tested by using an ELISA kit (R&D System, Minneapolis, MN) following the manufacturer’s instructions. The MIF concentration in the peritoneal lavage fluid or plasma of mice was tested by using another ELISA kit (BlueGene Biotech, China).

### Western blotting

For Western blot analysis, VE-cadherin (BD Biosciences, Franklin Lakes, NJ), p62 (Santa Cruz, Dallas, TX) and LC3 (GeneTex) were detected using a 1:1,000 dilution of antibodies followed by a 1:6,000 dilution of HRP-conjugated anti-mouse or anti-rabbit immunoglobulin antibody (Leadgene Biomedical). The β-actin (Table 1) antibody (Sigma-Aldrich) was used at a 1:10,000 dilution as an internal control. Bound HRP-conjugated antibodies were detected using the Luminata Crescendo Western HRP substrate (Merck Millipore, Germany). The Western blot results were quantified using the Image J software program.

**Table 1:** List of accession numbers for genes and proteins.

Gene	Description	SwissProt accession number
ACTB	β-actin	P60709
ATG5	Autophagy protein 5	Q9H1Y0
CDH5	VE-cadherin (CD144)	P33151
MAP1LC3B	Microtubule-associated proteins 1A/1B light chain 3B (LC3B)	Q9GZQ8
MIF	Macrophage migration inhibitory factor	P14174
SQSTM1	p62	Q13501

### Immunocytochemistry

Cell monolayers were seeded onto microscope cover glass. After treatment, the cells were fixed in 4% paraformaldehyde for 5 min, followed by three washes with PBS. The cells were then blocked with SuperBlock blocking buffer (Thermo Fisher Scientific) for 1 h at room temperature. To detect VE-cadherin and LC3 localization, specific antibodies against VE-cadherin (Beckman Coulter, Brea CA), and LC3 (Genetex) (1:200 dilutions in PBS) were incubated with cells overnight at 4°C. After three washes with tris-buffered saline and Tween 20, the cells were treated with an Alexa 488-conjugated goat anti-mouse IgG monoclonal antibody (Invitrogen, Carlsbad, CA) (1:500 dilution) and Alexa 594-conjugated goat anti-rabbit pAb (Invitrogen) (1:1,000 dilution) for 1 h, followed by three washes with tris-buffered saline-Tween 20. Images were obtained using a confocal microscope (Olympus FluoView FV1000, Melville, NY).

For quantifying barrier/marginal VE-cadherin, 3 μm across the cell border was defined as barrier/marginal area. And the remaining area within a cell was defined as cytosolic/perinuclear area. 50 cells in each condition were quantified by using Image J software.

### Statistical analysis

The data are expressed as the mean ± standard error of the mean (SEM) from more than three independent experiments. One-way ANOVA and Bonferroni's multiple comparison test as post-test, two-way ANOVA or Student’s *t*-test was used to analyze the significance of the difference between the test and the control groups by GraphPad Prism 5 software. *P* values < 0.05 were considered statistically significant.

## Supporting Information

S1 Fig Solvents, chemical drugs and antibodies alone have little effect on endothelial permeability. (A) HMEC-1 cells were treated with PBS, ISO-1, p425, 3-MA, NAC, anti-NS1 mAb 2E8, anti-NS1 pAb, anti-NS1 mAb DN5C6, or CTRL mIgG. The relative permeability of HMEC-1 cells was measured by RTCA every hour. n = 3, duplicated. (B) HMEC-1 cells were treated with PBS, DMSO, 20 μg/ml 293T-NS1, ISO-1, p425, anti-MIF pAb, 3-MA, NAC, or CTRL RaIgG. The volume of PBS was the same as that of 293T-NS1 and the volume of DMSO was the same as that of 3-MA in the vehicle controls. After 6 h, the relative permeability of HMEC-1 cells was measured by transwell assay. n = 3, triplicated. (C) HMEC-1 cells were treated with PBS, 20 μg/ml 293T-NS1, anti-NS1 mAb 2E8, anti-NS1 pAb, anti-NS1 mAb DN5C6, or CTRL mIgG. After 6 h, cell culture medium was collected, and MIF concentration was determined by ELISA. n = 3, triplicated.
(TIF)
