# Supplementary figures and images for: Dengue Virus Nonstructural Protein 1 Induces Vascular Leakage through Macrophage Migration Inhibitory Factor and Autophagy
Source: PLoS Negl Trop Dis. 2016 Jul 13;10(7):e0004828. doi: 10.1371/journal.pntd.0004828 (PMC4943727; doi:10.1371/journal.pntd.0004828)

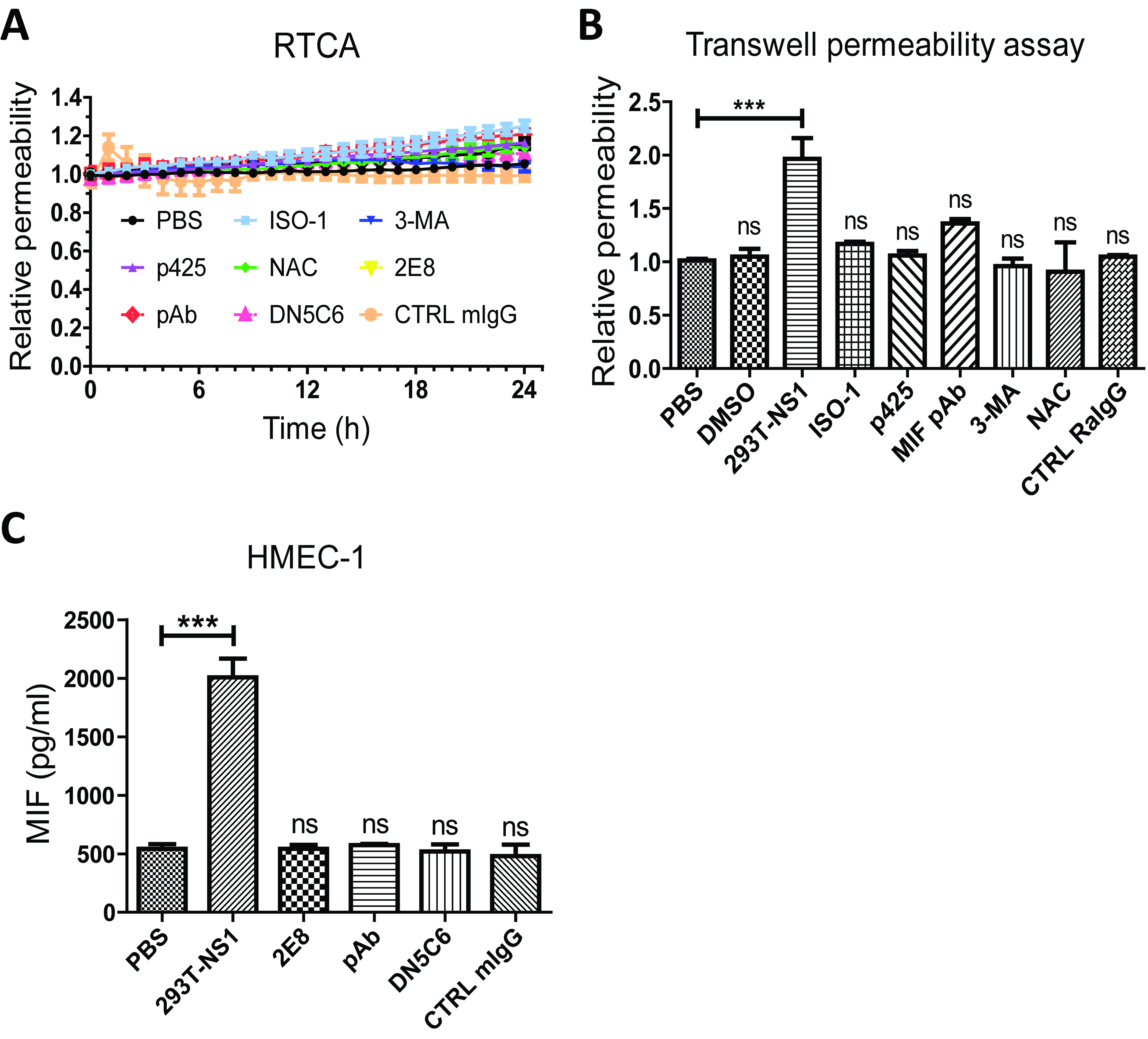

Supplement: S1 Fig — (A) HMEC-1 cells were treated with PBS, ISO-1, p425, 3-MA, NAC, anti-NS1 mAb 2E8, anti-NS1 pAb, anti-NS1 mAb DN5C6, or CTRL mIgG. The relative permeability of HMEC-1 cells was measured by RTCA every hour. n = 3, duplicated. (B) HMEC-1 cells were treated with PBS, DMSO, 20 μg/ml 293T-NS1, ISO-1, p425, anti-MIF pAb, 3-MA, NAC, or CTRL RaIgG. The volume of PBS was the same as that of 293T-NS1 and the volume of DMSO was the same as that of 3-MA in the vehicle controls. After 6 h, the relative permeability of HMEC-1 cells was measured by transwell assay. n = 3, triplicated. (C) HMEC-1 cells were treated with PBS, 20 μg/ml 293T-NS1, anti-NS1 mAb 2E8, anti-NS1 pAb, anti-NS1 mAb DN5C6, or CTRL mIgG. After 6 h, cell culture medium was collected, and MIF concentration was determined by ELISA. n = 3, triplicated. (TIF) [file pntd.0004828.s001.tif]
